# Prognostic factors of improvement in health-related quality of life in atomoxetine-treated children and adolescents with attention-deficit/hyperactivity disorder, based on a pooled analysis

**DOI:** 10.1007/s12402-013-0119-5

**Published:** 2013-10-20

**Authors:** Alonso Montoya, Deborah Quail, Ernie Anand, Esther Cardo, José A. Alda, Rodrigo Escobar

**Affiliations:** 1 Medical Neuroscience, Eli Lilly Canada Inc., 3650 Danforth Avenue, Toronto, ON M1N 2E8 Canada; 2 Lilly Research Centre, Windlesham, Surrey, UK; 3 Department of Pediatrics, Neuropediatric Hospital Son Llatzer, University of the Balearic Islands (UIB), Palma de Mallorca, Spain; 4 ADHD Unit, Department of Child and Adolescent Psychiatry and Psychology, University Hospital Sant Joan de Déu Barcelona, Barcelona, Spain; 5 Psychiatry and Pain Disorders, Eli Lilly and Company, Indianapolis, IN 46285 USA

**Keywords:** ADHD, Health-related quality of life, Atomoxetine, CHIP-CE, Response prediction

## Abstract

The objective of this study is to identify prognostic factors of treatment response to atomoxetine in improvement of health-related quality of life (HR-QoL), measured by the Child Health and Illness Profile-Child Edition Parent Report Form (CHIP-CE PRF) Achievement and Risk Avoidance domains, in children and adolescents with attention-deficit/hyperactivity disorder (ADHD). Pooled data from 3 placebo-controlled trials and separate data from 3 open-label trials of atomoxetine in children and adolescents with ADHD were analyzed using logistic regression methods. Based on baseline impairment in the Achievement and/or Risk Avoidance domains (CHIP-CE PRF < 40 points), 2 subsamples of subjects were included. Treatment outcome was categorized as <5 points or ≥5 points increase in the CHIP-CE PRF Achievement and Risk Avoidance domains. Data of 190 and 183 subjects from the pooled sample, and 422 and 355 subjects from the open-label trials were included in the analysis of Achievement and Risk Avoidance domains. Baseline CHIP-CE subdomain scores proved to be the most robust prognostic factors for treatment outcome in both domains, based on data from the pooled sample of double-blind studies and from the individual open-label studies (odds ratios [OR] 0.74–1.56, *p* < 0.05; OR < 1, indicating a worse baseline score associated with worse odds of responding). Initial treatment response (≥25 % reduction in ADHD Rating Scale scores in the first 4–6 weeks) was another robust prognostic factor, based on data from the open-label studies (OR 2.99–6.19, *p* < 0.05). Baseline impairment in HR-QoL and initial treatment response can be early prognostic factors of atomoxetine treatment outcome in HR-QoL in children and adolescents with ADHD.

## Introduction

Attention-deficit/hyperactivity disorder (ADHD) is a chronic neurodevelopmental disorder with core symptoms of inattention, hyperactivity, and impulsivity. ADHD is associated with significant impairment of cognitive, emotional, and psychosocial functioning (i.e., self-esteem, academic performance, and social acceptance, parent–child and family relationships), which goes beyond the core symptoms and has a strong impact on the patient’s health-related quality of life (HR-QoL) (Barkley 2002; Riley et al. 2006a; Escobar et al. 2005). HR-QoL is a concept that measures subjective perception of well-being in terms of physical, mental, and social domains (i.e., broader functioning), in addition to disease symptoms or treatment side effects. It is also a patient-centric (rather than clinician-centric) assessment (Escobar et al. 2010). For child or adolescent patients with ADHD, this is usually accomplished by measuring parents’ perceptions of the child’s well-being, although it is still considered to be more patient-centric than assessments by clinicians or biomedical measures.

Effective pharmacotherapeutic tools already exist for the treatment of ADHD (Nutt et al. 2007). Psychostimulants (Jensen et al. 2001) and atomoxetine are widely used and are recommended treatments for children and adolescents with ADHD (Cheng et al. 2007). The efficacy and tolerability of atomoxetine have already been demonstrated in a number of randomized, placebo-controlled trials (Michelson et al. 2001, 2002; Spencer et al. 2002) among children and adolescents. In addition, another body of literature reported on improvement of emotional well-being and HR-QoL in children and adolescents treated with atomoxetine (Michelson et al. 2001; Prasad et al. 2007), making atomoxetine the most extensively studied ADHD medication in terms of HR-QoL (Coghill 2010).

The Child Health and Illness Profile-Child Edition (CHIP-CE) (Riley et al. 2001) is a generic HR-QoL questionnaire covering 5 domains (Satisfaction, Comfort, Risk Avoidance, Resilience, and Achievement) and 12 subdomains (see Table 1). A pooled analysis of 5 non-US atomoxetine trials (*N* = 794) (Escobar et al. 2010) and a pan-European naturalistic study (Riley et al. 2006a), in which the CHIP-CE (Riley et al. 2001) was used for the assessment of HR-QoL, showed that the most severe and consistent baseline impairment in HR-QoL was present in the Achievement and Risk Avoidance domains (see Table 1). Additionally, based on the results of the pooled analysis, atomoxetine was predominantly effective in improving HR-QoL in the Achievement and Risk Avoidance domains (effect sizes 0.4 and 0.5, respectively) (Escobar et al. 2010).

**Table 1:** Child Health and Illness Profile-Child Edition—Parent Report Form (CHIP-CE PRF) domain and subdomain definitions (based on Riley et al. 2006b)

CHIP-CE domains and subdomains	Definition
Satisfaction domain	The parent’s assessment of the child’s sense of well-being and self-esteem (11 items)
Satisfaction with health	Overall perceptions of well-being and health
Satisfaction with self	General self-concept
Comfort domain	Parent’s assessment of the child’s experience of physical and emotional symptoms and positive health sensations and observed limitations of activity (22 items)
Physical comfort	Positive and negative somatic feelings and symptoms
Emotional comfort	Positive and negative emotional feelings and symptoms
Restricted activity	Restrictions in day-to-day activities due to illness
Resilience domain	Parent’s perception of the child’s participation in family, coping abilities, and physical activity (19 items)
Family involvement	Level of activities with family and perceived family support
Social problem solving	Active approaches to solving an interpersonal problem
Physical activity	Level of involvement in activities related to fitness
Risk Avoidance domain	Degree to which parent perceives that the child avoids behaviors that increase the likelihood of illness, injury, or poor social development (14 items)
Individual risk avoidance	Avoidance of activities that threaten individual health and development
Threats to achievement	Avoidance of behaviors that typically disrupt social development
Achievement domain	Extent to which the parent perceives that the child meets expectations for role performance in school and with peers (10 items)
Academic performance	School performance and engagement

Identification of prognostic factors of treatment response (e.g., HR-QoL criteria, biomarkers, neuroimaging) would be essential to individualize the optimal treatment for ADHD. Currently, there is a paucity of information about such possible factors of treatment response for ADHD medications in terms of improving HR-QoL.

The objective of this analysis was first to identify prognostic factors for treatment response to atomoxetine with regard to the improvement of HR-QoL, as measured by the CHIP-CE Achievement and Risk Avoidance domains, based on a pooled analysis of 3 placebo-controlled clinical atomoxetine trials conducted in children and adolescents with ADHD.

The secondary objective was to test whether the prognostic factors identified above were also predictive of response in further 3 open-label atomoxetine trials in children and adolescents with ADHD.

## Methods

### Studies included in the analysis

Pooled patient-level data from 3 double-blind, randomized, placebo-controlled trials and separate data from 3 open-label trials with similar inclusion and exclusion criteria were included in this analysis (Dickson et al. 2007; Prasad et al. 2007; Dell’Agnello et al. 2009; Escobar et al. 2009; Svanborg et al. 2009; Fuentes et al. 2010). A total of 1,192 patients were included in these studies. Design, sample size, and duration of the studies are described in Table 2.

**Table 2:** Basic information about the 6 clinical trials included in this meta-analysis

Study	Sample size (*N*) Male %	Design	Duration (weeks)	Dose (mg/kg/day)
Study 1 (S) Svanborg et al. (2009)	99 80.8	Randomized, double-blind, placebo-controlled	10	1.2
Study 2 (E) Escobar et al. (2009)	149 79.5	Randomized, double-blind, placebo-controlled	12	1.2
Study 3 (I) Dell’Agnello et al. (2009)	139 92.7	Randomized, double-blind, placebo-controlled	8	1.2
Study 4 (UK) Prasad et al. (2007)	201 88.6	Open-label, atomoxetine versus standard of care	10	0.5–1.8
Study 5 (CAN) Dickson et al. (2007)	206 74.1	Open-label, atomoxetine only	12	0.5–1.4
Study 6^a^ (EU, M) Fuentes et al. (2010)	398 79.4	Open-label, atomoxetine versus other early standard treatment	52	1.2–1.8

*CAN* Canada, *E* Spain, *EU* European Union, this study was conducted in 7 European countries (Spain, Belgium, UK, France, Turkey, Italy, Norway), *I* Italy, *M* Mexico, *S* Sweden, *UK* United Kingdom

^a^In case of Study 6, endpoint for this analysis was at week 16

Only non-US studies from the Lilly database were included, where a HR-QoL measure was used as primary/secondary objective, CHIP-CE was employed, main findings have been published, and data were available. These studies all had very similar designs, and all used the same HR-QoL outcome measures.

All included patients met the Diagnostic and Statistical Manual of Mental Disorders, Fourth Edition (DSM-IV-TR) (American Psychiatric Association 2000) diagnostic criteria for ADHD and had a symptom severity of at least 1.5 standard deviations (SDs) above the normative values of the Attention-Deficit/Hyperactivity Disorder Rating Scale–IV (ADHD-RS), Parent Version (DuPaul et al. 1998).

The following differences among the studies were noted:

Study 3, however, applied the ADHD subscale of the Swanson, Nolan, and Pelham-IV (SNAP-IV) (Swanson 1992).

In all studies with the exception of Study 5, the diagnosis was confirmed by the Kiddie Schedule for Affective Disorders and Schizophrenia for School Age Children-Present and Lifetime Version (K-SADS-PL) (Kaufman et al. 1997). The K-SADS-PL was also used for the assessment of comorbid psychiatric disorders (except for Study 3, in which SNAP-IV was applied for the evaluation of comorbid oppositional defiant disorder).

In Studies 2, 3, and 6, a baseline Clinical Global Impression of Severity (CGI-S) (Guy 1976) score of ≥4 was required for inclusion.

Studies 2 and 6 included medication-naïve patients only.

Study 3, which was conducted in Italy, did not explicitly require medication-naïve patients, but at the time of recruitment, there were no ADHD drugs approved by the authorities in Italy.

### Measures

#### CHIP-CE

This analysis was based on data assessed with the Child Health and Illness Profile-Child Edition Parent Report Form (CHIP-CE PRF) (Riley et al. 2001, 2006b), a 76-item generic HR-QoL questionnaire covering 5 domains (Satisfaction, Comfort, Risk Avoidance, Resilience, and Achievement) and 12 subdomains. Table 1 summarizes which aspects of HR-QoL are assessed by each domain and subdomain of the CHIP-CE.

CHIP-CE scores are standardized to t scores with a mean (±SD) of 50 (±10); higher scores indicate better health. Recently, the CHIP-CE total score was developed—this can be used as a global measure of HR-QoL (Riley et al. 2007).

#### WFIRS-P

The Weiss Functional Impairment Rating Scale-Parent Report (WFIRS-P) measures the impact of ADHD on the child’s functioning in multiple domains (Weiss and Weiss 2004) as rated by the parents. The 50-item WFIRS-P consists of 6 domains related to functioning: home, school, self-concept, social, activities of daily living, and risk taking. The WFIRS-P was applied as an additional QoL scale together with the CHIP-CE PRF in Studies 5 and 6 for assessing functional outcome.

### Statistical analysis

Two CHIP-CE domains (Achievement and Risk Avoidance) were considered in the analyses, and all analyses were conducted separately for each domain. Response was defined as a ≥5 points increase from baseline to endpoint in the domain score. We chose the 5 points improvement as response definition because it represents a 0.5 SD change in the CHIP-CE PRF, and a half SD difference is considered a clinically significant change in HR-QoL in a patient (Norman et al. 2003).

For the primary objective, data of all atomoxetine-treated patients from the 3 placebo-controlled trials were pooled. First, the 2 subpopulations of patients showing impairment in the examined domains of CHIP-CE (Achievement and Risk Avoidance) at baseline were identified. Impairment was defined as a baseline score of the respective domain <40. Data from the 2 subsamples (baseline impairment in the Achievement domain and baseline impairment in the Risk Avoidance domain) were analyzed separately.

For the purpose of identifying prognostic factors of treatment response to atomoxetine in the Achievement and Risk Avoidance domains of CHIP-CE (the dependent variables), logistic regression (binary logits) was performed for both domains. Possible prognostic factors included the following: study (membership in a certain study pooled for analysis), ADHD subtype (combined, hyperactive/impulsive, or inattentive), any preexisting psychiatric disorder (any affective, any anxiety, any tic disorders, oppositional defiant disorder/conduct disorder [ODD/CD], other), any early (first 2 weeks) treatment-emergent adverse events, age (subjects <12 or ≥12-year old), gender, race (Caucasian vs. other), baseline CHIP-CE subdomain scores, years since the onset of ADHD symptoms, ADHD-RS hyperactivity/impulsivity and inattentive subscores (subscales of SNAP-IV for Study 3), and baseline CGI-S. The full model was reduced by backward selection methods (i.e., going through iterations of excluding the least significant variable and refitting the model thereafter) until only explanatory variables statistically significant at the 5 % level remained. Model fit was assessed using the Hosmer–Lemeshow test (Hosmer and Lemeshow 2000). Adjusted odds ratios (ORs) and their 95 % confidence intervals (CIs) were calculated for each of the independent variables included in the reduced model. For the ‘CHIP-CE PRF baseline subdomain scores’ variable, ORs per −5 points are presented in order to show the change in odds associated with a clinically relevant worse score to ease interpretation.

For the secondary objective, the analyses above were repeated for each of the 3 open-label studies separately; only impaired subjects at baseline with regard to the Achievement and/or Risk Avoidance domains were included. In these analyses, additional independent variables were included where there were other potentially important measures collected. In all cases, initial symptomatic (treatment) response (defined as a 25 % decrease on the ADHD-RS total score during the first 4–6 weeks of the study) and, for Studies 5 and 6, WFIRS-P domain scores at baseline were also included as independent variables.

All tests of hypotheses were considered statistically significant if the 2-sided *p* value was ≤0.05. All analyses were done post hoc and are therefore exploratory.

## Results

### Patient population and disposition

The pooled sample from the 3 placebo-controlled trials included 255 patients who were randomized to atomoxetine treatment. Based on the subjects’ baseline impairment, analyses of the Achievement domain included 190 subjects (82.6 % male, 67.4 % <12-year old); analyses of the Risk Avoidance domain included 183 subjects (85.2 % male, 69.9 % <12-year old).

Baseline characteristics of the samples included in the analyses are summarized in Table 3.

**Table 3:** Baseline characteristics of the samples included in the analyses

Study CHIP-CE domain	Placebo-controlled studies Pooled sample (*N* = 255)^a^	Study 4 (*N* = 104)^a^	Study 5 (*N* = 221)^a^	Study 6 (*N* = 199)^a^
*Achievement domain*				
*n* (%)^b^	190 (74.5)	84 (80.8)	172 (77.8)	166 (83.4)
Male (%)	157 (82.6)	74 (88.1)	124 (72.1)	135 (81.3)
<12 years old (%)	128 (67.4)	53 (63.1)	172 (100)	136 (81.9)
ADHD subtype, *n* (%)				
Combined	149 (78.4)	75 (89.3)	137 (79.7)	131 (78.9)
Hyperactive/impulsive	7 (3.7)	2 (2.8)	3 (1.7)	3 (1.8)
Inattentive	34 (17.9)	7 (8.3)	32 (18.6)	32 (19.3)
Baseline score, mean (SD)^c^	28.0 (7.9)	23.6 (8.5)	28.1 (7.6)	24.6 (9.9)
Endpoint score, mean (SD)^c^	32.6 (9.7)	34.7 (13.1)	36.6 (10.8)	33.5 (13.0)
*Risk Avoidance domain*				
*n* (%)^b^	183 (71.8)	95 (91.3)	134 (60.6)	126 (63.3)
Male (%)	156 (85.2)	83 (87.3)	108 (80.6)	106 (84.1)
<12 years old (%)	128 (69.9)	61 (64.2)	134 (100)	104 (82.5)
ADHD subtype, *n* (%)				
Combined	155 (84.7)	85 (89.5)	115 (85.8)	108 (85.7)
Hyperactive/impulsive	8 (4.4)	2 (2.1)	3 (2.2)	2 (1.6)
Inattentive	20 (10.9)	8 (8.4)	16 (11.9)	16 (12.7)
Baseline score, mean (SD)^c^	27.4 (9.6)	16.4 (15.1)	26.8 (9.8)	17.8 (15.0)
Endpoint score, mean (SD)^c^	34.6 (10.7)	30.1 (16.9)	37.8 (12.3)	30.3 (19.4)

*ADHD* attention-deficit/hyperactivity disorder, *CHIP*-*CE PRF* Child Health and Illness Profile-Child Edition Parent Report Form, *SD* standard deviation

^a^Study population treated with atomoxetine of the respective studies

^b^Number of individuals in the sample with baseline impairment (CHIP-CE PRF Achievement domain/Risk Avoidance domain score <40). Rest of the data in this table refers to the impaired sample of the respective studies

^c^Baseline, endpoint in the CHIP-CE PRF Achievement domain/Risk Avoidance domain scores

### Prognostic factors: pooled data of the 3 placebo-controlled trials

#### Achievement domain

**Fig. 1:**
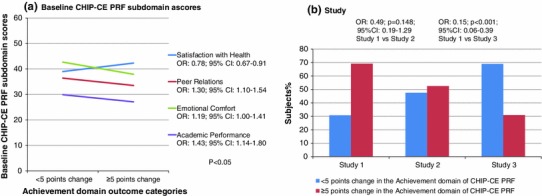
Achievement domain. **a** Prognostic factors found for the improvement in the Achievement domain of CHIP-CE PRF after atomoxetine treatment, based on pooled data of 3 double-blind placebo-controlled studies—baseline CHIP-CE PRF subdomains and **b** study (i.e., in which of the 3 original studies the subject participated). *CHIP*-*CE PRF* Child Health and Illness Profile-Child Edition Parent Report Form, *CI* confidence interval, *OR* odds ratio

The final model of the logistic regression included 5 variables, predicting treatment outcome with atomoxetine in the Achievement domain: (1) study (OR 0.15; *p* < 0.001; 95 % CI 0.06–0.39, Study 3 vs. Study 1). We found that individuals who were included in Study 1 had a higher chance of improving more than 5 points (0.5 SD) in the Achievement domain after treatment compared with those included in Study 3, but not compared with those in Study 2 (*p* = 0.15); (2) Academic Performance subdomain at baseline (OR 1.43; *p* = 0.002; 95 % CI 1.14–1.80); (3) Emotional Comfort subdomain at baseline (OR 1.19; *p* = 0.050; 95 % CI 1.00–1.41); (4) Peer Relations subdomain at baseline (OR 1.30; *p* = 0.002; 95 % CI 1.10–1.54); (5) Satisfaction with Health subdomain at baseline (OR 0.78; *p* = 0.001; 95 % CI 0.67–0.91). These results indicated that every −5 points (−0.5 SD) at baseline in the Academic Performance, Emotional Comfort, and Peer Relations subdomains increased the odds for improving more than 5 points in the Achievement domain after treatment. In case of the Satisfaction with Health subdomain, results showed that the more impaired the subject was at baseline, the less improvement could be observed in the Achievement domain after the treatment (Fig. 1).

#### Risk Avoidance domain

**Fig. 2:**
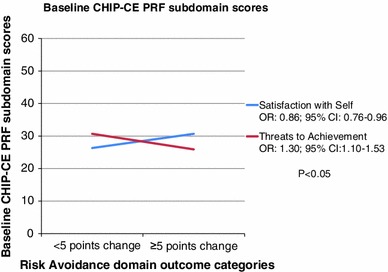
Risk Avoidance domain: Prognostic factors found for the improvement in the Risk Avoidance domain of CHIP-CE PRF after atomoxetine treatment, based on pooled data of 3 double-blind placebo-controlled studies—baseline CHIP-CE PRF subdomains. *CHIP*-*CE PRF* Child Health and Illness Profile-Child Edition Parent Report Form, *CI* confidence interval, *OR* odds ratio

The final logistic regression model included 2 prognostic factors of treatment outcome with atomoxetine: (1) Satisfaction with Self subdomain at baseline (OR 0.86; *p* = 0.009; 95 % CI 0.76–0.96), indicating that the more impaired at baseline the subject was, the less chance for improvement could be expected in the Risk Avoidance domain after treatment; (2) Threats to Achievement subdomain at baseline (OR 1.30; *p* = 0.002; 95 % CI 1.10–1.53), showing that the more impaired the subject was at baseline, the higher the chance was to improve more than 5 points in the Risk Avoidance domain after treatment (Fig. 2).

### Prognostic factors: open-label studies

Table 4 summarizes the final models by CHIP-CE PRF outcome domains (Achievement, Risk Avoidance), including prognostic factor variables for each study separately.

**Table 4:** Prognostic factors of treatment response to atomoxetine in CHIP-CE PRF Achievement and Risk Avoidance domains, based on data of the 3 open-label trials

Study	CHIP-CE PRF outcome domain	Predictor	OR	*p* value	95 % CI
Study 4	Achievement domain	None	NA	NA	NA
	Risk avoidance domain	Initial response (yes vs. no)	2.99	0.038	1.06–8.43
		Individual risk avoidance^a^	1.27	0.016	1.05–1.55
Study 5	Achievement domain	Age (years)	1.42	0.016	1.07–1.90
		Initial response (yes vs. no)	3.11	0.018	1.22–7.95
		Academic performance^a^	1.56	0.001	1.19–2.06
		Individual risk avoidance^a^	1.23	0.028	1.02–1.48
		Physical activity^a^	1.52	<0.001	1.20–1.92
		Satisfaction with health^a^	0.74	0.018	0.58–0.95
		Social problem solving^a^	0.77	0.006	0.64–0.93
		CGI-S at baseline	0.56	0.039	0.32–0.97
		WFIRS-P activities of daily living subscore^b^	0.70	0.009	1.54–0.91
		WFIRS-P self-concept subscore^b^	1.38	0.033	1.02–1.85
	Risk Avoidance domain	Race (Caucasian vs. other)	5.15	0.026	1.21–21.89
		Initial response (yes vs. no)	6.19	<0.001	2.38–16.11
Study 6	Achievement domain	Age (years)	0.75	0.015	0.59–0.95
		Gender (male vs. female)	0.18	0.003	0.06–0.56
		Years since onset of ADHD symptoms	1.47	0.002	1.16–1.86
		Peer relations^a^	1.28	0.004	1.08–1.52
		WFIRS-P social activities subscore^b^	0.70	0.008	0.53–0.91
	Risk Avoidance domain	Age (years)	0.71	0.017	0.54–0.94
		Years since onset of ADHD symptoms	1.48	0.008	1.11–1.97
		WFIRS-P social activities subscore^b^	0.64	<0.001	0.50–0.81

*ADHD* attention-deficit/hyperactivity disorder, *CGI*-*S* Clinical Global Impression of Severity, *CHIP*-*CE PRF* Child Health and Illness Profile-Child Edition Parent Report Form, *CI* confidence interval, *NA* not applicable, *OR* odds ratio, *WFIRS*-*P* Weiss Functional Impairment Rating Scale-Parent Report

^a^Subdomain baseline score, OR is given by every −5 points at baseline

^b^Subscore at baseline. For the WFIRS-P domain scores, OR by half a standard deviation increase is presented

Baseline CHIP-CE PRF and WFIRS-P subdomain scores were shown to be prognostic factors for both Achievement and Risk Avoidance domains. The direction of the prediction was generally that there is a higher chance for a better outcome when more severe baseline impairment is observed (OR 1.23–1.56). However, there are CHIP-CE PRF/WFIRS-P subdomains that showed the opposite: CHIP-CE PRF Satisfaction with Health, Social Problem Solving subdomains, WFIRS-P Activities of daily living, Self-concept subscores in Study 5, and WFIRS-P Social activities subscore in Study 6.

Among the open-label studies, initial treatment response and age were also shown to be relatively robust prognostic factors of HR-QoL outcome. Odds ratios for initial treatment response were as follows: 3.11 with regard to the Achievement domain (Study 5), and ranging between 2.99 and 6.19 with regard to the Risk Avoidance domain (Studies 4 and 5, respectively); ORs for age were as follows: 1.42 for the Achievement domain in Study 5, and 0.75 and 0.71 (*p* < 0.05) for the Achievement and the Risk Avoidance domains in Study 6, respectively.

Additionally, in Study 6, gender and years since onset of ADHD symptoms were also identified as prognostic factors. In specific, female gender and more years with ADHD were prognostic for better improvement.

In the case of Study 5, we considered age as a continuous variable since there were no subjects >12-year old included in this study. In the case of Study 6, after the initial analysis, we found that age (as a categorical variable: subjects <12 or ≥12-year old) was included in the final model (OR 12.1; 95 % CI 2.5–58.6) of the Risk Avoidance domain as a prognostic factor. To test the robustness of this finding, we reran the analysis with age as a continuous variable for both domains. Table 4 includes the findings of this second analysis. Based on the first analysis of Study 6 data, the model for the Achievement domain included comorbid ODD/CD (OR 2.85; 95 % CI 1.17–6.92) and CHIP-CE PRF Threats to Achievement baseline score (OR 0.81; 95 % CI 0.69–0.96). Both variables disappeared from the model during the second analysis. Age, years since onset of ADHD, and WFIRS-P Social activities subscore were not in the first model, but were included in the second model of the Achievement domain. Prognostic factors included in the final model for Risk Avoidance domain did not change after the second analysis.

## Discussion

Across the samples of double-blind and open-label studies of atomoxetine, one common pattern was that baseline impairment in HR-QoL (as measured by the CHIP-CE PRF and/or WFIRS-P) could predict the outcome in Achievement and Risk Avoidance domains. Although the predictive subdomains of CHIP-CE PRF/WFIRS-P were different across studies, 2 repeating patterns could be observed. First, more severe baseline impairment in the subdomains of the respective outcome domain (Achievement or Risk Avoidance) was a general prognostic factor for better outcome. This finding is in line with expectations, since the analyses were not controlled for the baseline scores of the examined outcome domains themselves. Second, while in most cases, lower baseline subdomain scores of CHIP-CE (indicating more severe impairment) predicted a higher chance for a better outcome, even having adjusted for the effect of these subdomains, for the Satisfaction with Health, Satisfaction with Self, and Social Problem Solving subdomains, an opposite direction of prediction could be observed. Specifically, lower baseline scores in these latter subdomains (as well as in the WFIRS-P Social activities subscore) predicted less improvement in the Achievement/Risk Avoidance domains. We hypothesize that this finding can be explained, at least in part, by the clinical and empirical observation that satisfied, optimistic patients respond better to treatment compared with those with negative thoughts, chronic pessimistic viewpoints, and lack of satisfaction about themselves (in regard to their condition or in general).

Another robust finding of this analysis was that in the open-label studies (Studies 4 and 5), initial treatment response in ADHD core symptoms predicted improvement in both Achievement and Risk Avoidance domains. Specifically, subjects showing at least a 25 % decrease in the ADHD-RS score during the first 4–6 weeks of treatment had a higher chance for improvement in the Achievement/Risk Avoidance domains of CHIP-CE as well. This finding is in line with findings in the literature showing consistent associations between improvement in the core symptoms of ADHD and improvement in QoL scores, with minimal or no time lag in studies with both methylphenidate and atomoxetine (Coghill 2010; Weiss et al. 2010). It must be noted that HR-QoL and core symptoms are overlapping but distinct concepts. Studies have already demonstrated that the therapeutic response in core symptoms does not fully explain the response with regard to HR-QoL (Escobar et al. 2010).

Unfortunately, not all of the double-blind studies included the variable initial treatment response, and thus, it was impossible to use this variable as a possible prognostic factor in the analysis of the pooled sample of the double-blind studies.

Further findings based on pooled data from the double-blind studies showed that those subjects who participated in Study 1 had higher chance for improvement in the Achievement domain compared with those included in Study 3, but not compared with those in Study 2. When interpreting this finding, we must note that there were 2 main differences across samples of these studies. First, in Study 3, only ADHD + ODD patients participated; second, Study 1 included a 4-week parental psychoeducation intervention (2 h weekly). Both differences might be taken into account in the background of the above finding. The positive role of psychoeducational interventions has already been shown in outcome measures, such as treatment response, in children and adolescents with ADHD (Montoya et al. 2011). Regarding the comorbidity with ODD, the pertinent literature suggests that children and adolescents with ADHD and comorbid ODD/CD show more HR-QoL impairment (Riley et al. 2006a; Coghill 2010). Up until now, the predictive role of comorbid ODD with ADHD in terms of treatment outcome—either negative or positive—has not been clarified (Ollendick et al. 2008).

In a recent publication, Wehmeier et al. (2010), using pooled data from 5 of the 6 atomoxetine studies included in this analysis, reported that in regard to the Risk Avoidance domain, adolescents might benefit more from atomoxetine treatment than children. Adolescents also showed more clinically relevant improvement in the Achievement domain as well. According to the authors, this difference may not have reached statistical significance because of the small sample size of adolescents (Wehmeier et al. 2010). Based on our present analyses, age did not prove to be a consistent prognostic factor of improvement either in the Achievement or Risk Avoidance domain. The findings of our analyses with regard to age as a possible prognostic factor are further complicated by another finding, specifically, that in Study 6, more years since onset of ADHD symptoms was shown to be a prognostic factor for better improvement in both outcome domains. One would think that older individuals have more years since onset of ADHD symptoms. However, as was already presented, according to the findings in Study 6, younger subjects would benefit more from treatment with atomoxetine with regard to HR-QoL.

To the authors’ knowledge, this analysis is the first to directly examine possible prognostic factors for atomoxetine treatment outcome with regard to HR-QoL in children and adolescents with ADHD. Other studies have examined the effect of quality of life in children/adolescents receiving other pharmacotherapies for ADHD, but these data were not available to us when the atomoxetine HR-QoL studies were designed, and those studies used other HR-QoL instruments as well as varying study designs. Nonetheless, the pool of HR-QoL data is larger for atomoxetine than for other ADHD treatments, and we are not aware of any other analyses like ours with those other compounds.

The role of HR-QoL in understanding disease progression or predicting treatment outcomes is gaining greater attention. Studies have shown a uniform pattern of change in HR-QoL with ADHD treatment, with improvements in HR-QoL occurring concurrent with symptom improvement in both children/adolescents and adults (Frazier et al. 2010; Weiss et al. 2010). Thus, HR-QoL improvement does not appear to be a delayed response to symptom improvement with treatment.

### Limitations

Our findings need to be interpreted in light of certain limitations. First, although the studies included in this analysis had similar inclusion and exclusion criteria, as well as duration and medication doses applied, heterogeneity still appears across studies in terms of the sample and methodology. This makes it difficult to compare findings from the individual studies. It must be noted, however, that none of the included studies had been originally designed to test those specific questions we aimed to investigate in our post hoc analysis. Second, the pooled sample as well as the individual open-label studies contained predominantly boys and children, leaving only a small sample of girls and adolescents (especially adolescent girls). Finally, ADHD is a chronic disorder, and our conclusions can only be drawn for the length of the clinical studies, reflecting the available data.

## Conclusion

This analysis used a broad approach to investigate HR-QoL treatment outcome with atomoxetine in children and adolescents with ADHD.

Based on our findings, baseline impairment in HR-QoL domains and initial treatment response in terms of ADHD core symptoms seem to be predictive of HR-QoL treatment outcome with atomoxetine in the case of the Achievement and Risk Avoidance domains, as measured by the CHIP-CE. Studies directly targeting the identification of prognostic factors of improvement in HR-QoL in children and adolescents with ADHD are needed to help clinical practitioners to make individual therapeutic decisions.
